# Dramatic niche shifts and morphological change in two insular bird species

**DOI:** 10.1098/rsos.140364

**Published:** 2015-03-04

**Authors:** Per Alström, Knud A. Jønsson, Jon Fjeldså, Anders Ödeen, Per G. P. Ericson, Martin Irestedt

**Affiliations:** 1 Swedish Species Information Centre, Swedish University of Agricultural Sciences, PO Box 7007, Uppsala 75007, Sweden; 2 Key Laboratory of Zoological Systematics and Evolution, Institute of Zoology, Chinese Academy of Sciences, 1 Beichen West Road, Chaoyang District, Beijing 100101, People's Republic of China; 3 Center for Macroecology, Evolution and Climate at the Natural History Museum of Denmark, University of Copenhagen, Copenhagen, Denmark; 4 Department of Life Sciences, Imperial College London, Silwood Park Campus, Ascot SL5 7PY, UK; 5 Department of Life Sciences, Natural History Museum, Cromwell Road, London SW7 5BD, UK; 6 Department of Animal Ecology, Uppsala University, Norbyvägen 18D, Uppsala 75236, Sweden; 7 Department of Zoology, and, Swedish Museum of Natural History, PO Box 50007, Stockholm 10405, Sweden; 8 Department of Bioinformatics and Genetics, Swedish Museum of Natural History, PO Box 50007, Stockholm 10405, Sweden

**Keywords:** speciation, adaptive change, niche shifts, morphological divergence

## Abstract

Colonizations of islands are often associated with rapid morphological divergence. We present two previously unrecognized cases of dramatic morphological change and niche shifts in connection with colonization of tropical forest-covered islands. These evolutionary changes have concealed the fact that the passerine birds madanga, *Madanga ruficollis*, from Buru, Indonesia, and São Tomé shorttail, *Amaurocichla bocagii*, from São Tomé, Gulf of Guinea, are forest-adapted members of the family Motacillidae (pipits and wagtails). We show that *Madanga* has diverged mainly in plumage, which may be the result of selection for improved camouflage in its new arboreal niche, while selection pressures for other morphological changes have probably been weak owing to preadaptations for the novel niche. By contrast, we suggest that *Amaurocichla*'s niche change has led to divergence in both structure and plumage.

## 2. Introduction

Colonizations of islands are well-known triggers of speciation [1–4], often involving strong morphological divergence from continental relatives, sometimes resulting in spectacular radiations [5–9]. Such morphological divergence may result in erroneous assumptions about systematics as well as misinterpretations of biogeographical and ecological patterns and processes (cf. [10–16]).

We examine the phylogenetic relationships of two enigmatic forest birds of tropical islands, madanga *Madanga ruficollis* from Buru, Wallacea, Indonesia, and São Tomé shorttail *Amaurocichla bocagii* from São Tomé in the Gulf of Guinea, and demonstrate that both have undergone remarkable morphological change and niche shifts from their continental relatives, completely obscuring their systematic relationships. *Madanga ruficollis* is known from four old museum specimens [17] and a few recent observations [18–20], and has traditionally been placed in a monotypic genus in the family Zosteropidae (‘white-eyes’) [21,22]. *Amaurocichla bocagii* is placed in a monotypic genus, with presumed sylvioid relationships [23,24], although recent molecular analysis unexpectedly suggested affinities to *Motacilla* [25].

## 3. Material and methods

### 3.1 Study group

For a first preliminary molecular analysis of *Madanga*, we used mainly GenBank sequences (electronic supplementary material, table S1), representing a broad array of lineages, including most of the primary lineages within Passerida, as suggested by previous studies (e.g. [25–27]). As we obtained strong support for a close relationship between *Madanga* and Motacillidae, as had previously been suggested also for *Amaurocichla* [25], we expanded the dataset to also include representatives from all genera within Motacillidae and multiple species from the main clades found in earlier studies [28,29]. When preliminary analyses suggested a relationship between *Madanga* and a clade referred to as ‘small pipits’ by Alström & Mild [30], all the species in that clade were analysed (except *Anthus petrosus*, which has previously been treated as conspecific with *Anthus spinoletta*; cf. [30], as well as the two remaining Asian species with untested relationships, alpine pipit *Anthus gutturalis* and Nilgiri pipit *Anthus nilghiriensis*.

### 3.2 DNA extraction and sequencing

DNA was obtained from muscle, blood or, in a few cases, feathers or toepads (electronic supplementary material, table S1). Toepads were sampled from two *Madanga* and two *Amaurocichla* specimens. While standard laboratory procedures were used for fresh DNA samples, extractions, amplifications and sequencing procedures from archaic DNA obtained from study skin samples followed the procedures described previously [31,32]. This included extracting DNA in a dedicated ‘clean’ laboratory, and amplifying short (*ca* 200 bp), partly overlapping fragments. We sequenced five loci: the main part of the mitochondrial cytochrome *b* gene and part of the flanking tRNA-Thr (combined referred to as *cytb*); the mitochondrial NADH dehydrogenase subunit 2 (*ND2*); the nuclear ornithine decarboxylase (*ODC*) exon 6 (partial), intron 6, exon 7, intron 7 and exon 8 (partial); the entire nuclear myoglobin (*myo*) intron 2 and the Z-linked (*CHD1Z*) intron.

### 3.3 Phylogenetic analyses

In addition to the sequences obtained specifically for this study, we also used sequences from GenBank (electronic supplementary material, table S1). *Cytb* and *ND2* were analysed for all species and two to three nuclear loci for most species. For *Anthus spragueii* only *Cytb* was available, and for *Anthus brachyurus* only *cytb* and *ND2*. See electronic supplementary material, table S1 for details. All new sequences have been deposited in GenBank (electronic supplementary material, table S1).

Sequences were aligned using Muscle [33] in Seaview v. 4.3.4 [34,35]; some manual adjustment was carried out for the non-coding sequences. For the nuclear loci, heterozygous sites were coded as ambiguous. Trees were estimated by Bayesian inference (BI) using MrBayes v. 3.2 [36,37] as follows. (i) For the Motacillidae dataset, using *Emberiza* and *Passer* as outgroups, all loci and all sequences, including multiple individuals of the same species when available, were analysed separately (single-locus analyses). (ii) For the same dataset, as well as the one comprising multiple oscine lineages, using the suboscines *Tyrannus* and *Manacus* as outgroups, sequences from one individual per species were concatenated, partitioned by locus and, for the coding loci, in two codon partitions (1*st*+2*nd* and 3rd). Partitioning schemes and models were selected based on the Bayesian Information Criterion calculated in PartitionFinder v. 1.1.1 [38]: for *cytb* and *ND2* 1*st*+2*nd* codon partitions, the HKY model [39], assuming rate variation across sites according to a discrete gamma distribution with four rate categories (*Γ* [40]) and an estimated proportion of invariant sites (I [41]); for *cytb* and *ND2* 3rd codon positions, the general time-reversible (GTR) model [42–44] + *Γ*; and for the other partitions, *HKY*+*Γ*. Rate multipliers were applied to allow different rates for different partitions [37,45]. Ambiguous base pairs and indels were treated as missing data, but indels were plotted on the trees *a posteriori*. Default priors in MrBayes were used. Four Metropolis-coupled MCMC chains with incremental heating temperature 0.1 or 0.05 were run for 3×10^6^ (single-locus analyses) or 5×10^6^ (multilocus analyses) generations and sampled every 1000 generations. Convergence to the stationary distribution of the single chains was inspected in Tracer v. 1.5.0 [46] using a minimum threshold for the effective sample size. The joint likelihood and other parameter values reported large effective sample sizes (more than 1000). Good mixing of the MCMC and reproducibility was established by multiple runs from independent starting points. Topological convergence was examined by eye and by the average standard deviation of split frequencies (less than 0.005). The first 25% of generations were discarded as ‘burn-in’, well after stationarity of chain likelihood values had been established, and the posterior probabilities were calculated from the remaining samples (pooled from the two simultaneous runs).

A chronogram was reconstructed for the *cytb* data in BEAST v. 1.7.5 [47,48]. The topology was constrained to match the multilocus tree. XML files were generated in BEAUti v. 1.7.5 [49]. Analyses were run under the *GTR*+*Γ* model (cf. [50]), using a ‘birth–death incomplete sampling’ prior, and (i) a fixed clock rate of 2.1%/Myr [50] or (ii) an uncorrelated lognormal relaxed clock [51] with the same mean rate. Other priors were used with default values. For these analyses, 50×10^6^ generations were run, sampled every 1000 generations. Every analysis was run twice. The MCMC output was analysed in Tracer v. 1.5.0 [46] to evaluate whether valid estimates of the posterior distribution of the parameters had been obtained. The first 25% of the generations were discarded as ‘burn-in’, well after stationarity of chain likelihood values had been established. Trees were summarized using TreeAnnotator v. 1.7.5 [52], choosing ‘Maximum clade credibility tree’ and ‘Mean heights’, and displayed in FigTree v. 1.3.1 [53].

The single-locus and concatenated data were also analysed by maximum-likelihood bootstrapping (MLBS). RAxML-HPC2 v. 7.3.2 [54,55] was run on the Cipres portal [51], 1000 replicates. The data were partitioned by locus, and GTRGAMMA was used both for the bootstrapping phase and for the final tree inference.

### 3.4 Morphological analyses

We examined all four known specimens of *Madanga* and four specimens of *Amaurocichla* in the American Museum of Natural History, New York (AMNH) and one additional specimen (holotype) of *Amaurocichla* in The Natural History Museum, Tring, UK (NHM). These were compared with large series of pipits (*Anthus*) and wagtails (*Motacilla*) and other relevant species. All the species in clade A (see Results) were measured: wing length (flattened and stretched); tail length (ruler inserted under tail); bill length (to skull); bill depth and width (at distal edges of nostrils); tarsus length (to last complete scutum before toes); hind-claw length (to thin skin at base). Only internally sexed specimens were measured, with the aim to measure 10 of each sex when possible. Measurements were taken in the AMNH, Swedish Museum of Natural History, Stockholm (NRM) and National Zoological Museum of China, Beijing (NZMC). A principal component analysis (PCA) was performed in SPSS Statistics v. 20 (IBM Corp.). In addition, X-rays of all four *Madanga* specimens were examined and compared with skeletons of other relevant taxa.

## 4. Results

### 4.1 Molecular markers

**Figure 1.**
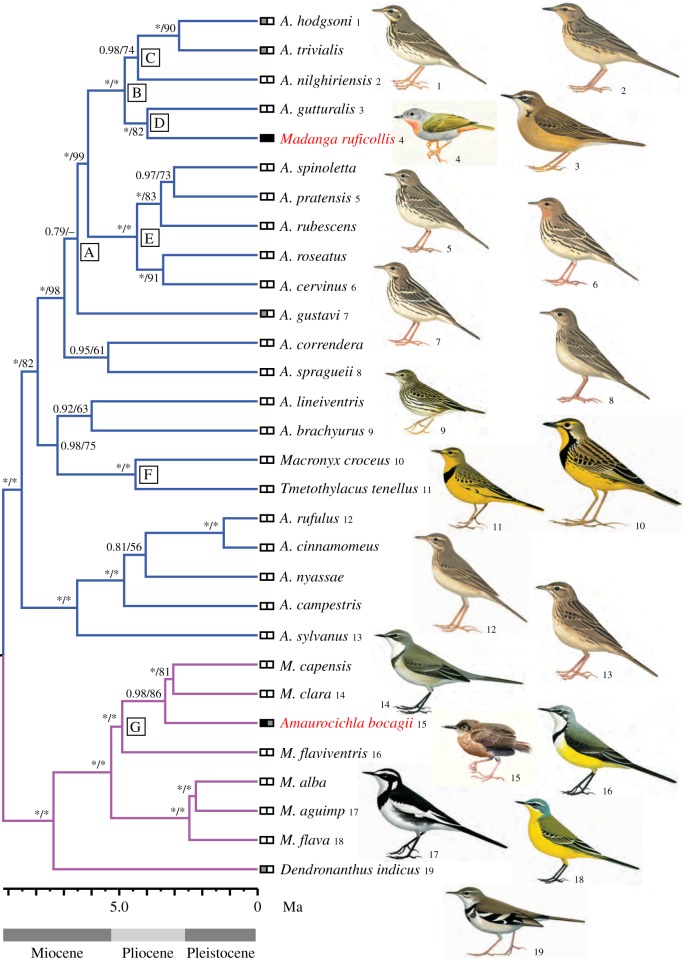
Chronogram of Motacillidae, based on *cytb* and a molecular clock (2.1%/Myr), with the topology constrained to fit the multilocus tree (electronic supplementary material, figure S2). Values at branches are posterior probabilites (PP)/maximum-likelihood bootstrap (MLBS) percentages; *> indicates PP 1.00 or MLBS 100%. Letters A–G denote clades discussed in the text. The ‘pipit’ and ‘wagtail’ clades have been highlighted by differently coloured branches; *A.—Anthus*, *M.—Motacilla*. Boxes at tips of branches represent, from left to right, habitat (black—forest; white—open; grey—forest or more open) and foraging niche (black—arboreal; white—terrestrial; grey—terrestrial and arboreal). Illustrations by P.A. (from [30]), Ren Hathway (3, 9–11, 14, 16–19; from [56]) and J.F. (4, 15).

*Madanga* was nested within Motacillidae, among the primarily Eurasian ‘small pipits’ *Anthus* (*sensu* [30]) (figure 1, clade A; electronic supplementary material, figures S1–S3). *Madanga* and the New Guinean *A. gutturalis* formed a strongly supported clade (D), sister to another well supported clade (C) comprising the northern Palaearctic breeders *A. trivialis* and *A. hodgsoni* and South Indian *A. nilghiriensis*. *Amaurocichla* was nested within *Motacilla*, as sister to the Afrotropical *M. clara* and *M. capensis*, in a clade (G) also containing the Malagasy *M. flaviventris*; these clades were strongly supported. Clade D was also supported by a unique 19 bp deletion in the ODC alignment (electronic supplementary material, figure S2). In the single-locus analyses (electronic supplementary material, figure S3), there was conflict between mitochondrial and nuclear markers regarding the exact position of *Madanga*, with the mitochondrial tree inferring *Madanga* to be sister to the four other species in clade B. There was also some conflict among single-locus analyses regarding the precise position of *Amaurocichla* (electronic supplementary material, figure S3). Although not the focus of this study, *Macronyx croceus* and *Tmetothylacus tenellus* were inferred to be sisters (clade F), deeply nested within *Anthus*, and *Dendronanthus indicus* was found to be sister to *Motacilla*; all these relationships received strong support.

*Madanga* and *A. gutturalis* were estimated to have diverged from a most recent common ancestor 3.98 (95% HPD 2.65–5.33) million years ago (Ma), *Amaurocicha* and *M. capensis*+*M. clara* 3.34 (95% HPD 2.17–4.64) Ma (figure 1; electronic supplementary material, figure S4).

### 4.2 Morphology

**Figure 2.**
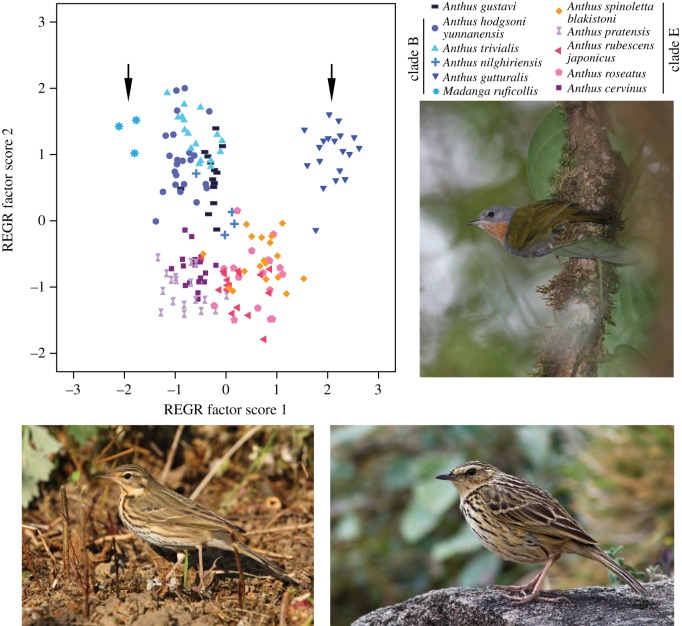
Principal component analysis of seven structural variables of the ‘small pipits’ *sensu* [30]. Arrows indicate *Madanga ruficollis* and its sister *Anthus gutturalis*. Photos of *M. ruficollis* (top) and two pipits (*Anthus hodgsoni yunnanensis*, bottom left; *Anthus nilghiriensis*) in their typical habitats. Photos Rob Hutchinson, Aurélien Audevard and Arka Sarkar, respectively.

*Madanga* shows hardly any plumage similarity with *Anthus* (e.g. completely lacks dark streaking; shows unpatterned head, with no pale supercilium or dark moustachial and malar stripes; rather uniformly green wings without contrastingly pale wing-bars or tertial edgings; vivid green upperparts, and grey head and underparts with a well-demarcated pale rufous throat patch; figure 1). The only decidedly *Anthus*-like plumage trait is the pale markings on the inner webs of the outermost pair of rectrices, although these are less contrasting than in *Anthus*. By contrast, *Madanga* displays typical *Anthus* structure, although it is smaller than any Holarctic/Oriental *Anthus*, with less elongated tertials; on regression factor 1, which reflects overall size, *Madanga* is completely separated from the other ‘small pipits’ (figure 2). It most resembles *A. hodgsoni* and *A. trivialis*, especially in bill and hind-claw structure (figure 2; electronic supplementary material, tables S2–S3). By contrast, *Madanga*'s sister, *A. gutturalis*, is the largest species in clade A, although it resembles *Madanga* in bill and hind-claw structure (figure 2; electronic supplementary material, tables S2–S3).

*Madanga* differs from all species in Zosteropidae by its rufous throat and in multiple structural aspects (e.g. proportionately longer toes and claws, and the presence of a slight hump on the culmen of the upper mandible over the elliptical nostrils; by contrast, zosteropids have an evenly curved culmen to the upper mandible and slit-like nostrils with more pronounced operculum).

The plumage of *Amaurocichla* bears no resemblance to any *Motacilla*, being uniformly dark brown above and on the wings and tail and paler brown below with slightly paler upper throat and belly (figure 1). Moreover, the blackish bill often has diffusely set-off paler tip, unlike all wagtails. Also the structure is strongly divergent from all wagtails, having proportionately longer bill, much shorter, more rounded wings and much shorter tertials and tail, with 10 rectrices (12 in *Motacilla*) with projecting shafts (figure 1). *Amaurocichla* differs markedly in structure from the ‘warbler’ genus *Macrosphenus*, with which it has been associated [24], by e.g. its vestigial 10th primary, slight hump at the base of the culmen of the upper mandible, no hook at the tip of the upper mandible, less prominent rictal bristles, and proportionately longer and slimmer tarsi, toes and claws, the latter also less strongly decurved.

## 5. Discussion

### 5.1 Island colonizations, niche shifts and morphological divergence

We provide evidence that two motacillid species have successfully colonized tropical islands, Buru and São Tomé, although they have diverged so much in morphology, habitat choice and behaviour (cf. figure 1) that their systematic affinities have been gravely misinterpreted. Despite the genus *Anthus*, with more than 40 extant species [22], having existed for some 8–9 Myr (electronic supplementary material, figure S4), the plumage divergence within this genus has been minimal compared to most other similar sized groups of birds, and different pipit species are renowned for being difficult to separate morphologically [30,56,57]. By stark contrast, Madanga's plumage does not resemble that of any other pipit. Likewise, *Amaurocichla bocagii* is very different from other wagtails in plumage and has also diverged dramatically in bill, wing and tail structure.

Colonization of islands requires the capacity to cross open water and may be further facilitated by sociality, which increases the potential for simultaneous arrival of multiple individuals [4]. The family Motacillidae is globally distributed, and includes several long-distance migrants with loose flocking behaviour [28,30,56,58], making them suitable candidates for island colonization. Indeed, endemic pipits are found on several oceanic islands, and one species complex has colonized multiple islands from Sri Lanka to New Zealand [56].

*Madanga* occurs in stunted montane forest [19,59], where it has been noted to feed like a nuthatch (*Sitta*) on epiphyte-covered branches and tree trunks, sometimes following mixed-species feeding flocks [59] (R. Hutchinson, F. Rheindt, P.-H. Fabre 2013, personal communication). *Amaurocichla* is found in primary forest, where descriptions in the literature suggest diverse foraging strategies, including feeding on the ground in riparian habitats as well as on tree trunks and branches [24,60,61]. In contrast to both *Madanga* and *Amaurocichla*, nearly all other motacillids occur in open habitats with short grass, such as savannah, steppe, meadows and tundra, and along rivers and lake sides, from sea level to above the tree limit [30,56]. Moreover, unlike *Madanga* and *Amaurocichla*, all other motacillids forage exclusively on the ground [30,56] (the statement that *Dendronanthus indicus* ‘is equally likely to seek food in trees [as on the ground]’ [56] is incorrect [30]; personal observation).

The radical niche shifts in *Madanga* and *Amaurocichla* were most likely instigated by their colonization of tropical islands that, before human settlement, were probably completely forest-covered. In the case of *Madanga*, the conditions for a niche shift were probably favourable, as its ancestor, by chance, settled in an area with strongly epiphyte-covered, i.e. unusually ‘ground-like’, trees (by contrast, its sister species, *A. gutturalis*, colonized an area with open grasslands [56], i.e. a common pipit habitat). We hypothesize that *Madanga*'s niche shift originated by opportunistic feeding in trees used for shelter, and that it was morphologically and behaviourally suitably preadapted for this new niche. For example, short, decurved hind-claws, as in all species in clade B, are typical of arboreal birds (cf. the longer, straighter hind-claws of the species in clade E [30]); two of *Madanga*'s closest relatives, *A. trivialis* and *A. hodgsoni*, are atypical among pipits in breeding mainly in wooded habitats, and in frequently taking cover in trees when flushed off the ground [30,56], and *Madanga*'s poorly known sister species, *A. gutturalis*, is also said to often fly into bushes and trees when alarmed [56]; the species in clade C, which is sister to the *Madanga/ A. gutturalis* clade, forage by creeping around in rather dense vegetation of grasses and forbs [30], a habitat rather similar to epiphyte-covered branches; and the species in clade C are all capable of walking rather freely on branches [30]. Novel feeding behaviours leading to niche shifts might spread quickly in a small founder population, first culturally and later obtaining a genetic basis (cf. [4, pp. 128, 133]).

*Amaurocichla*'s niche shift is less marked than in the case of *Madanga*, as it is less arboreal. Its close relative *M. flaviventris* sometimes breeds in forest clearings, open secondary forest and *Eucalyptus* plantations, and the even more closely related *M. clara* occurs along streams and rivers in forested country (although not in closed forest) [56,62].

In general, birds show a strong correlation between morphology and ecology (review [63]). Niche shifts can result in feeding-related morphological differentiation, as in the Hawaiian honeycreepers [8,64], Malagasy vangas [9,65], Darwin's finches (e.g. [6]) and ground tit *Pseudopodoces humilis* [66]. This seems to be true also for *Amaurocichla*, which has diverged markedly in structure from other motacillids, which are all structurally basically similar. *Amaurocichla*'s notable bill elongation is almost certainly feeding-related, whereas the dramatically shortened tail and wings and change in plumage are likely adaptations to its closed forest habitat. Shortening of the tail and wings and cryptic plumage colouration would be advantageous in closed forest, and short tails and wings and mostly brown plumage are common in various distantly related groups of passerines inhabiting dense undergrowth (e.g. many Cettiidae, Pellorneidae, Pnoepygidae, Troglodytidae and Rhinocryptidae) (e.g. [67,68]).

In the case of *Madanga*, surprisingly little divergence has occurred in feeding-related morphology, despite the extraordinary switch in niche and substantial period (*ca* 4 Myr) of independent evolution (cf. *Amaurocichla*, which has been separated from its closest relatives for 3.3 Myr). We suggest that the selection for feeding-related morphological change has been weak because the lineage leading to *Madanga* was suitably preadapted to this new niche (see above). However, the smaller size, which is the main structural difference between *Madanga* and its relatives, might have evolved in response to the niche change, as a smaller, lighter body would be advantageous for feeding on branches and trunks. By contrast, the plumage might have been under strong selection for improved crypsis in its novel niche. At least the plain green upperparts of *Madanga* probably provide better camouflage in trees than the contrastingly marked upperparts of most other pipits, as indicated by the fact that uniformly green upperparts are common in arboreal passerines in many unrelated families (e.g. Pycnonotidae, Phylloscopidae, Zosteropidae, Regulidae, Chloropseidae and Vireonidae). It is possible that *Madanga*'s strong plumage divergence is at least partly the result of changed sexual selection pressures, though the data are not available to test this. A similar example, with strong plumage and niche divergence but comparatively slight structural differentiation, concerns the cinnamon ibon *Hypocryptadius cinnamomeus*, also previously misclassified as an aberrant zosteropid but now identified as a canopy-adapted sparrow [13].

### 5.2 Taxonomy

A comprehensive taxonomic revision of Motacillidae is required. As a first step, *Madanga* should be synonymized with *Anthus* (type species: *A. pratensis*) and *Amaurocichla* with *Motacilla*. However, we advocate awaiting a more comprehensive sampling before making any further taxonomic recommendations.

### 5.3 Conclusion

To conclude, we suggest that the strong morphological divergences in the lineages leading to *Madanga ruficollis* and *Amaurocichla bocagii* were triggered by fundamental niche shifts following colonizations of forest-covered tropical islands.

## Supplementary Material

Fig. S1. MB Madanga Passeriformes AllLoci 4parts Jan 2014-2.pdf multilocus tree of Passeriformes

## Supplementary Material

Fig. S2. MB Madanga All Loci 7 p 131116.pdf - multilocus tree of Motacillidae

## Supplementary Material

Fig. S3. Madanga Fig. S3 Single-locus analyses.pdf - single locus analyses

## Supplementary Material

Fig. S4. BEAST Madanga Cytb ShortInkAspr LogNorm2,1%.pdf - Chronogram

## Supplementary Material

Table S1 Madanga Samples.xlsx - table with sample information

## Supplementary Material

Madanga ESM Revised 5 Oct 2014.docx - Supplementary Table S2 and Supplementary figure captions

## Supplementary Material

Table S3 PCA Madanga.pdf - PCA output

## Data accessibility

Supplementary figures and tables, DNA sequence alignments and details of samples, including GenBank numbers (KP671495–KP671599) and photos are deposited in Dryad: http://dx.doi.org/10.5061/dryad.40s3g.
