# Supplementary figures and images for: Dramatic niche shifts and morphological change in two insular bird species
Source: R Soc Open Sci. 2015 Mar 4;2(3):140364. doi: 10.1098/rsos.140364 (PMC4448822; doi:10.1098/rsos.140364)

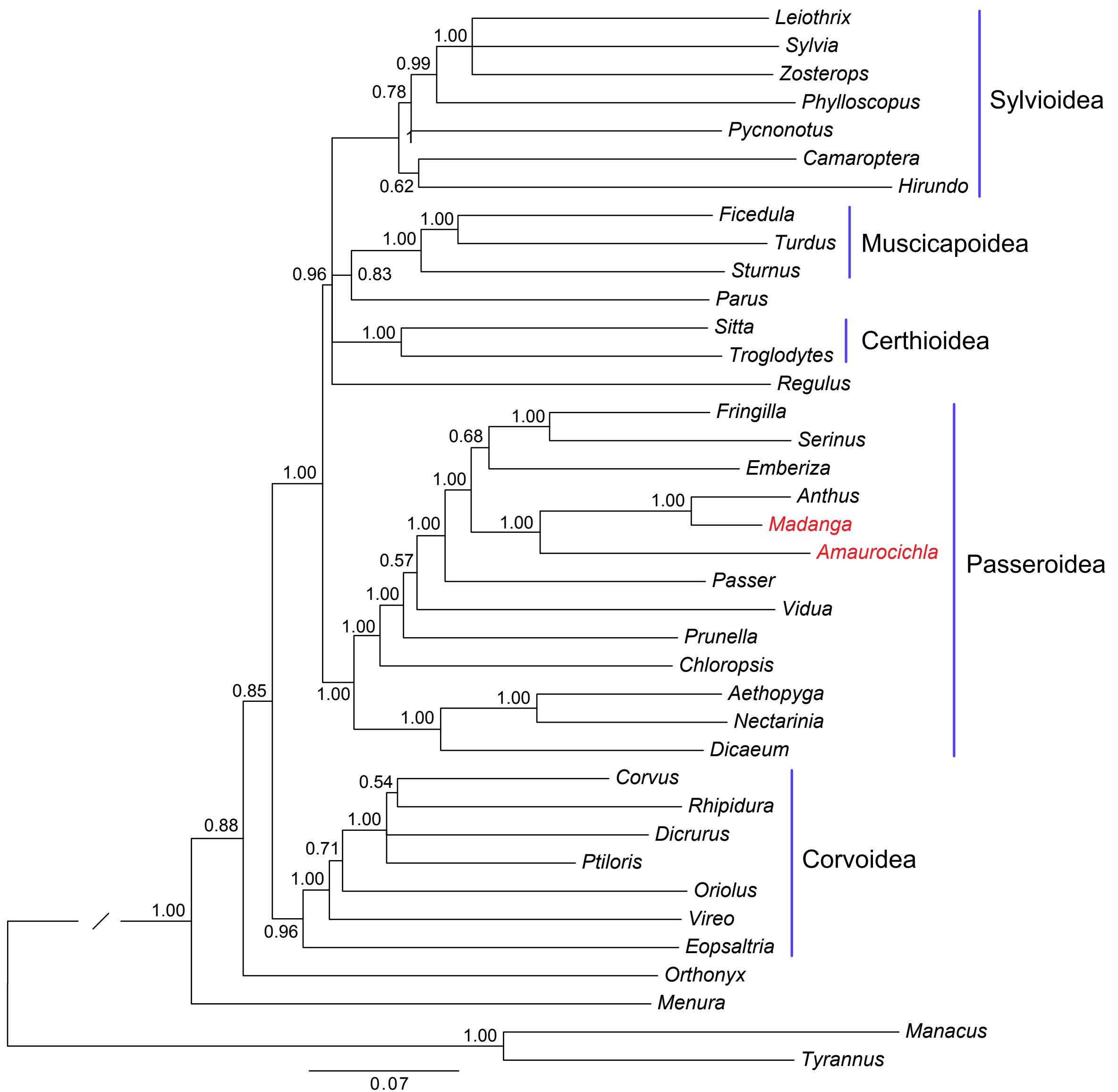

Supplement: Fig. S1. MB Madanga Passeriformes AllLoci 4parts Jan 2014-2.pdf multilocus tree of Passeriformes [file rsos140364supp1.pdf]

All loci  
(7 partitions)

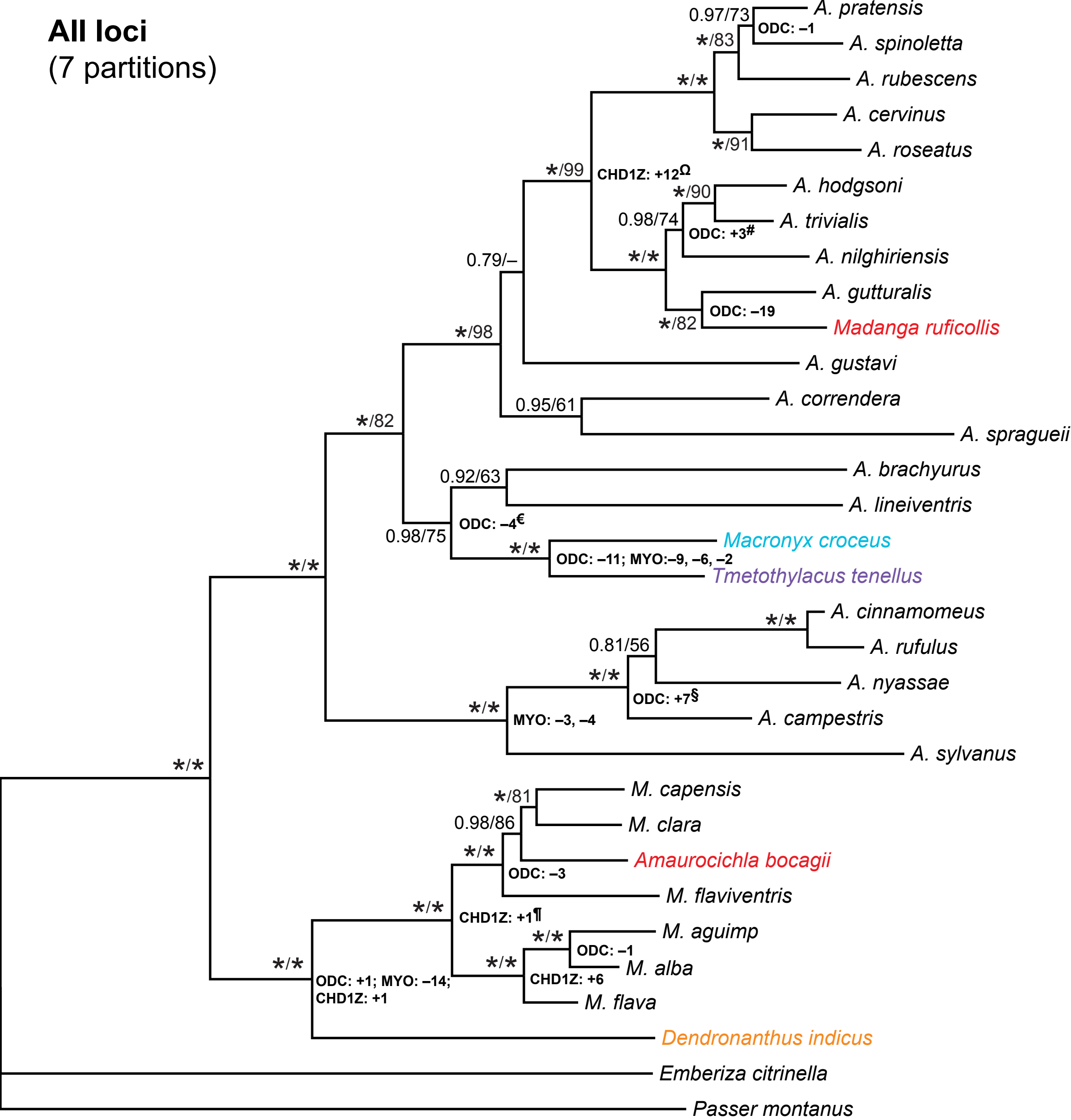

Supplement: Fig. S2. MB Madanga All Loci 7 p 131116.pdf - multilocus tree of Motacillidae [file rsos140364supp2.pdf]

# ND2 + Cytb (4 partitions)

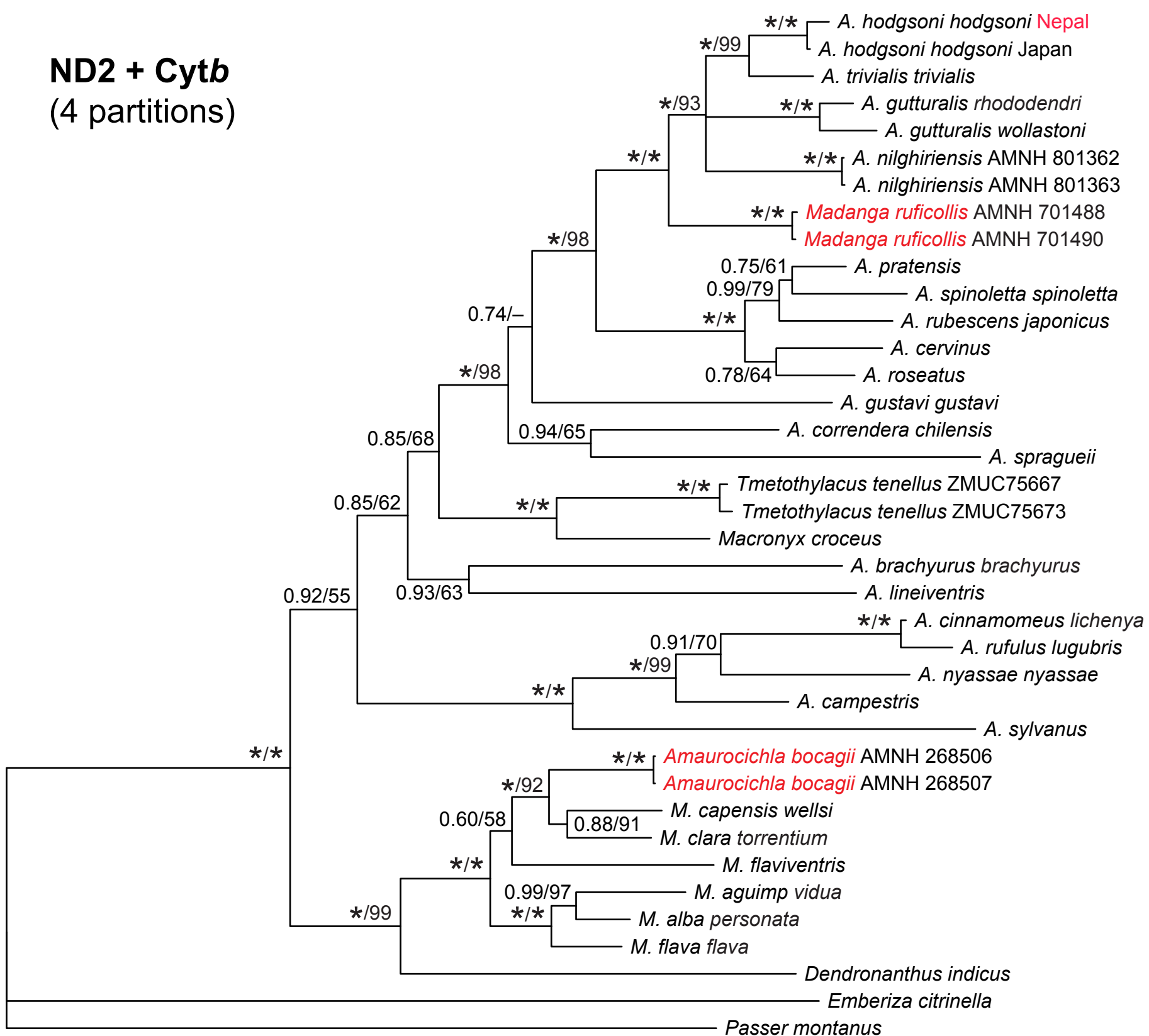

# Myoglobin

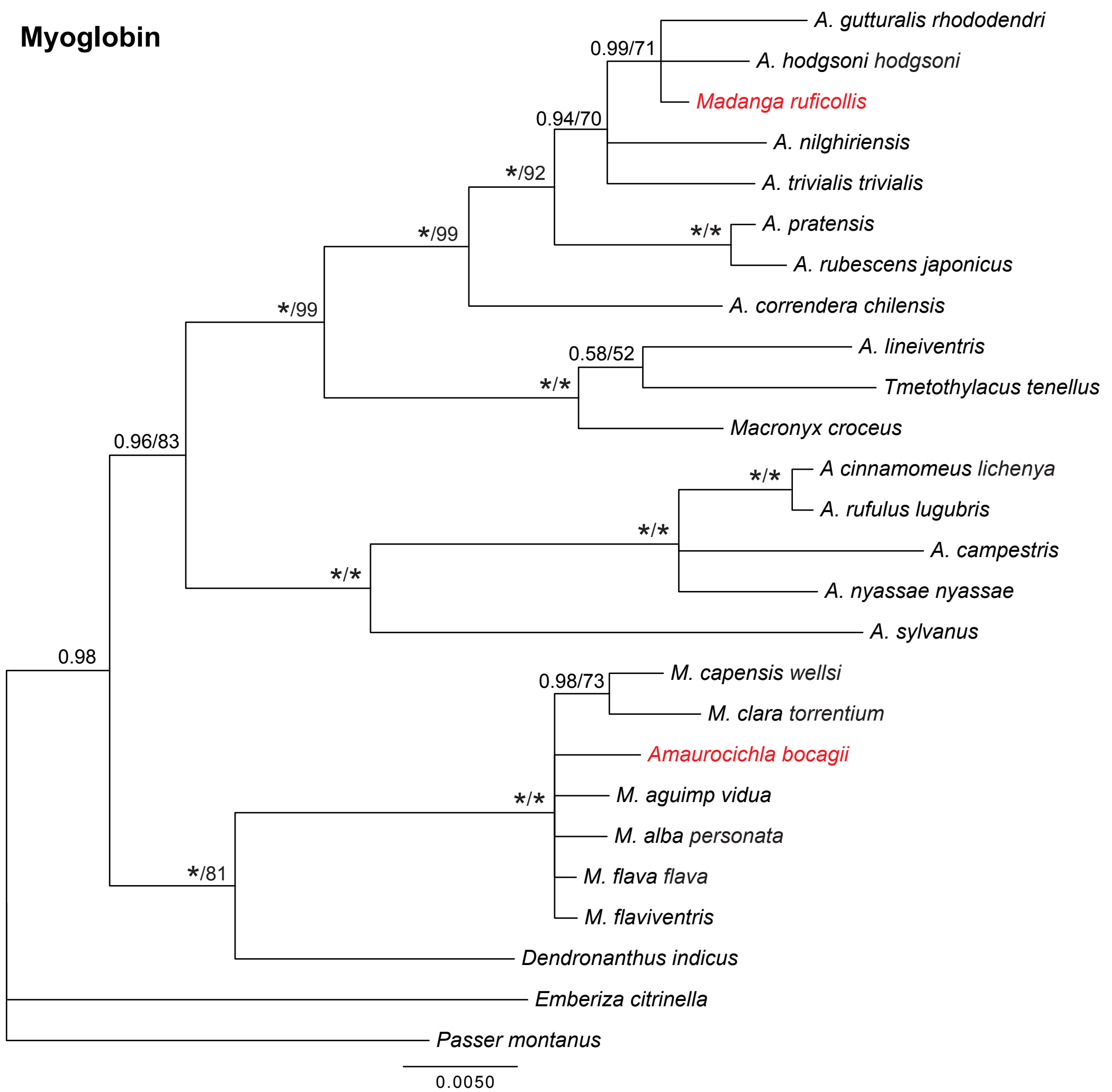

# ODC

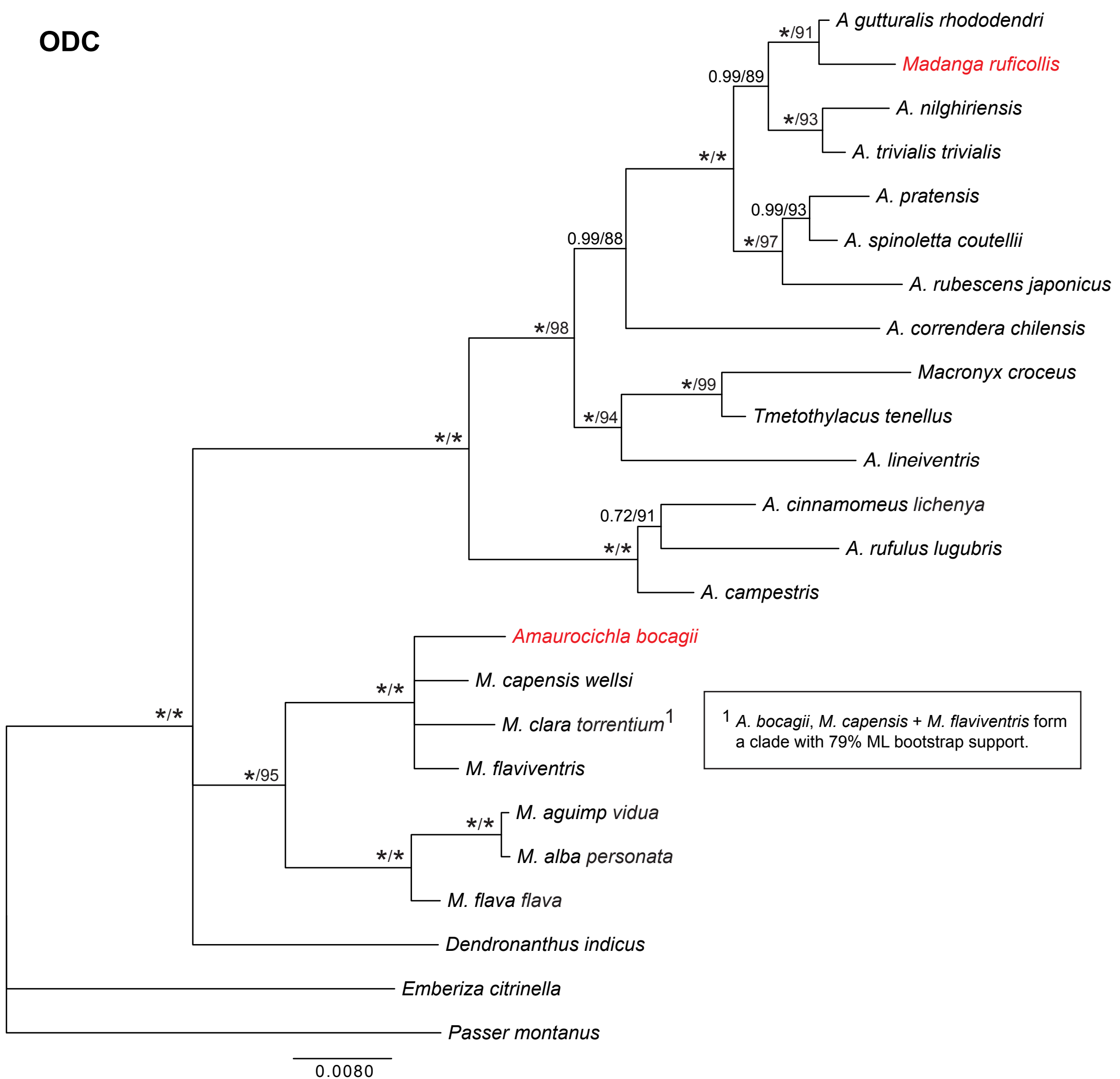

# CHD1Z

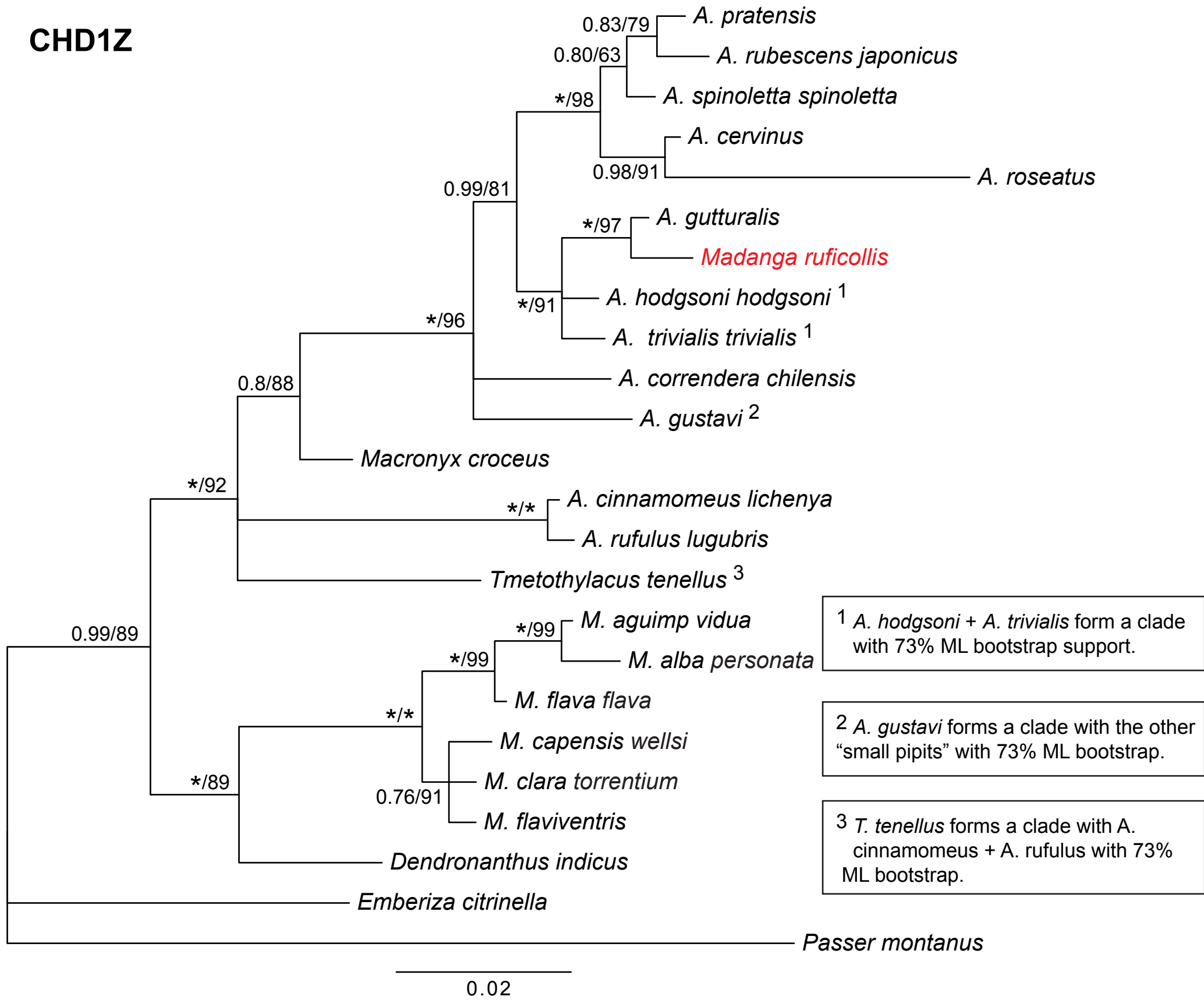

Supplement: Fig. S3. Madanga Fig. S3 Single-locus analyses.pdf - single locus analyses [file rsos140364supp3.pdf]

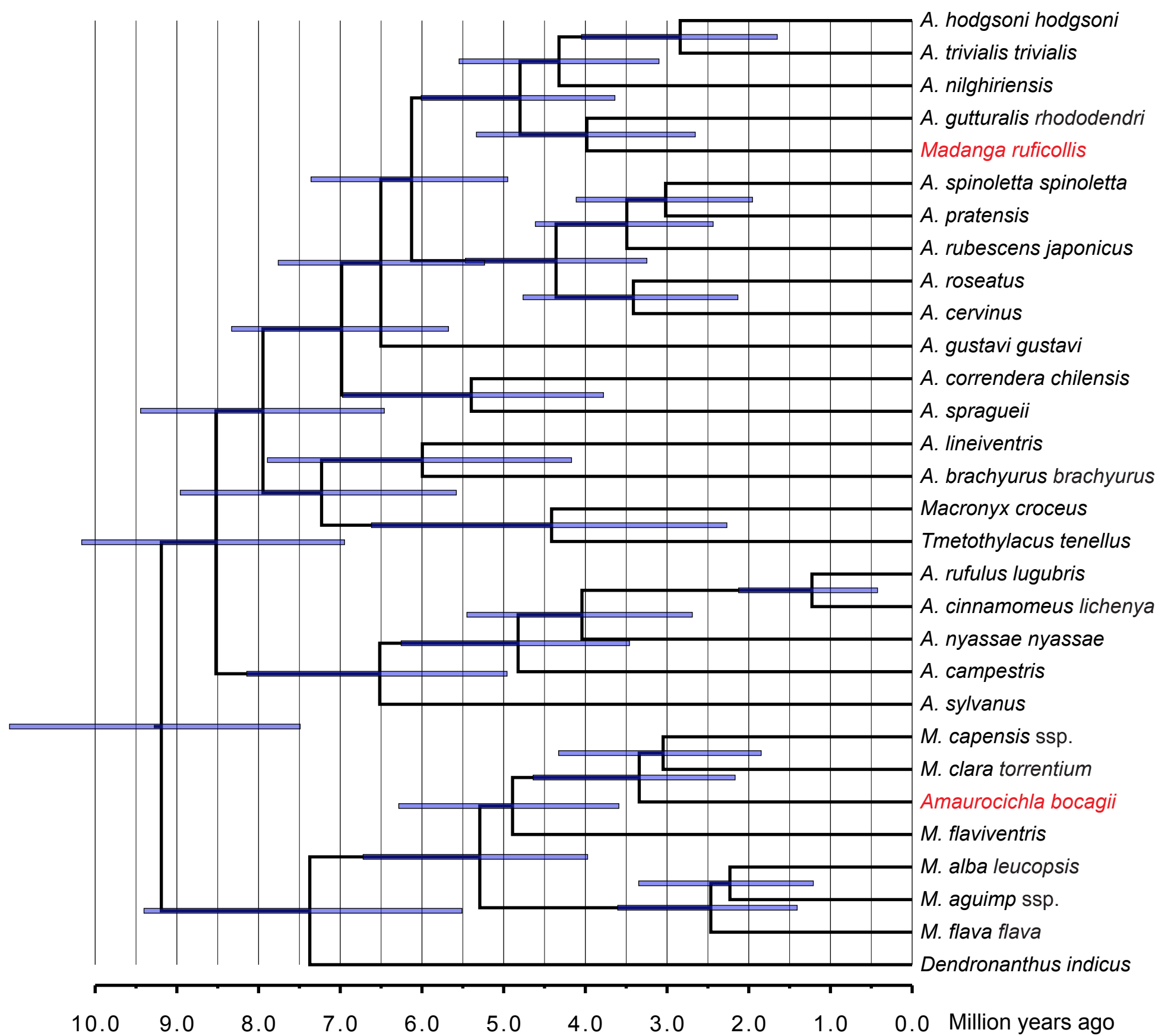

Supplement: Fig. S4. BEAST Madanga Cytb ShortInkAspr LogNorm2,1%.pdf - Chronogram [file rsos140364supp4.pdf]
