# Supplementary material for: Dramatic niche shifts and morphological change in two insular bird species
Source: R Soc Open Sci. 2015 Mar 4;2(3):140364. doi: 10.1098/rsos.140364 (PMC4448822; doi:10.1098/rsos.140364)
Supplement: Madanga ESM Revised 5 Oct 2014.docx - Supplementary Table S2 and Supplementary figure captions [file rsos140364supp6.docx]

|  | **wing** | **tail** | **bill length** | **bill depth** | **bill width** | **tarsus** | **hind claw** |
| --- | --- | --- | --- | --- | --- | --- | --- |
| *Madanga ruficollis* AMNH701487 (ht) M | 75 | 54,5 | 14,9 | 3,5 | 3,7 | 19,7 | 7,2 |
| *Madanga ruficollis* AMNH701490 (pt) F | 71 | 49,5 | 15 | 3,4 | 3,6 | 20 | 7,3 |
| *Madanga ruficollis* Unsexed (2) | 72.3; 0.4; 72.0–72.5 | 53 (N=1) | 15.5; 0.1; 15.4–15.5 | 3.3; 0.1; 3.2–3.3 | 3.5; 0.1; 3.4–3.6 | 19.9; 0.1; 19.8–20.0 | 7.1; 0.3; 6.9–7.3 |
| *Anthus gutturalis* M (10) | 100.8; 2.2; 97.0–104.5 | 75.0; 1.7; 71.0–78.5; | 18.1; 0.8; 16.0–19.0 | 4.1; 0.1; 3.9–4.3 | 3.9; 0.2; 3.5–4.4 | 28.5; 0.8; 26.3–29.4 | 8.7; 0.4; 8.2–9.5 |
| *Anthus gutturalis* F (6) | 96.7; 1.9; 94.0–99-0 | 73.0; 2.3; 71.0–77.0 | 18.3; 0.5; 17.9–18.9 | 4.0; 0.3; 3.4–4.3 | 4.0; 0.2; 3.6–4.3 | 28.1; 0.8; 27.1–29.2 | 8.7; 0.4; 8.2–9.4 |
| *Anthus nilghiriensis* M (3) | 79.8; 1.6; 78.0–81.0 | 64.0; 1.0; 63.0–65.0 | 16.2; 0.4; 15.9–16.6 | 3.7; 0.1; 3.6–3.8 | 3.3; 0.2; 3.2–3.6 | 24.2; 0.8; 23.3–24.8 | 10.4; 0.2; 10.2–10.5 |
| *Anthus nilghiriensis* F (1) | 73.5 | 60.0 | 15.4 | 3.9 | 3.3 | 25.5 | 9.0 |
| *Anthus hodgsoni yunnanensis* M (10) | 85.7; 1.5; 83.0–88.5 | 59.8; 2.4; 54.0–62.5 | 15.4; 0.6; 14.5–16.2 | 3.6; 0.4; 3.2–4.3 | 3.4; 0.2; 3.2–3.7 | 20.6; 0.8; 19.2–20.1 | 7.6; 0.5; 6.6–8.1 |
| *Anthus hodgsoni yunnanensis* F (0) | 82.5; 1.4; 81.0–85.0 | 57.1; 2.2; 54.0–60.0 | 15.2; 0.6; 14.1–16.1 | 3.6; 0.2; 3.3–3.9 | 3.4; 0.3; 2.8–3.9 | 20.5; 0.9; 19.3–21.6 | 8.1; 0.5; 6.9–8.8 |
| *Anthus roseatus* M (10) | 93.8; 1.5; 91.5–96.0 | 67.5; 2.1; 64.0–72.0 | 16.4; 0.7; 14.9–17.2 | 3.3; 0.2; 2.9–3.4 | 3.1; 0.2; 2.8–3.4 | 22.8; 0.8; 21.0–24.1 | 9.8; 1.1; 7.6–11.2 |
| *Anthus roseatus* F (5) | 86.8; 2.3; 84.5–90.0 | 59.1; 1.8; 56.5–61.0 | 16.0; 0.4; 15.6–16.5 | 3.2; 0.2; 3.0–3.3 | 3.1; 0.1; 3.0–3.3 | 22.2; 0.5; 21.7–22.9 | 11.1; 0.7; 10.2–12.0 |
| *Anthus rubescens japonicus* M (10) | 88.8; 2.6; 90.0–92.0 | 63.8; 2.0; 60.5–67.0 | 16.0; 0.6; 15.2–16.8 | 3.1; 0.2; 2.8–3.4 | 3.1; 0.2; 2.9–3.3 | 22.2; 0.9; 20.5–23.3 | 10.3; 0.9; 9.2–12.2 |
| *Anthus rubescens japonicus* F (8) | 85.6; 2.5; 82.0–89.5 | 62.2; 3.5; 57.0–68.0 | 15.8; 0.5; 14.8–16.4 | 3.1; 0.2; 2.9–3.3 | 3.1; 0.2; 2.9–3.3 | 22.2; 0.4; 21.4–22.6 | 10.7; 1.0; 9.1–12.5 |
| *Anthus gustavi* M (10) | 85.5; 1.5; 83.5–88.5 | 53.7; 1.4; 51.0–56.0 | 16.6; 0.4; 16.0–17.3 | 3.9; 0.3; 3.2–4.2 | 3.6; 0.3; 3.0–4.0 | 22.9; 0.6; 22.0–23.8 | 10.8; 0.6; 9.7–12.1 |
| *Anthus gustavi* F (4) | 82.5; 2.3; 79.5–84.5 | 52.4; 1.9; 50.5–55.0 | 16.7; 0.8; 15.5–17.2 | 3.7; 0.2; 3.5–3.9 | 3.7; 0.3; 3.4–4.0 | 22.4; 0.4; 21.9–22.8 | 10.8; 0.5; 10.3–11.5 |
| *Anthus pratensis* M (10) | 81.1; 2.1; 79.0–85.5 | 58.8; 2.0; 55.0–62.0 | 14.8; 1.0; 12.9–16.3 | 3.1; 0.2; 2.8–3.3 | 2.9; 0.1; 2.6–3.1 | 20.4; 0.6; 19.0–20.8 | 11.0; 0.5; 10.1–11.7 |
| *Anthus pratensis* F (8) | 78.1; 2.0; 76.0–81.5 | 55.8; 2.6; 52.0–59.5 | 14.7; 0.7; 13.7–15.6 | 3.1; 0.2; 2.8–3.3 | 3.0; 0.2; 2.7–3.2 | 20.2; 0.6; 19.2–21.3 | 10.7; 0.5; 10.3–11.7 |
| *Anthus trivialis* M (10) | 90.0; 1.7; 87.0–93.5 | 61.9; 2.2; 59.0–65.0 | 15.4; 0.5; 14.6–16.2 | 3.8; 0.1; 3.7–4.0 | 3.6; 0.2; 3.3–3.8 | 20.8; 0.8; 19.6–22.3 | 7.8; 0.4; 7.3–8.7 |
| *Anthus trivialis* F (6) | 86.4; 1.6; 84.0–88.0 | 60.3; 1.2; 58.0–61.0 | 15.1; 0.6; 14.4–15.9 | 3.8; 0.2; 3.5–3.9 | 3.8; 0.2; 3.6–4.0 | 19.9; 0.3; 19.5–20.5 | 7.7; 0.5; 7.1–8.6 |
| *Anthus cervinus* M (10) | 85.5; 1.6; 83.0–88.0 | 57.8; 1.0; 56.0–59.5 | 14.7; 0.4; 14.0–15.4 | 3.1; 0.1; 3.0–3.3 | 3.2; 0.1; 3.0–3.3 | 21.0; 0.5; 20.3–21.7 | 10.8; 0.9; 8.9–12.6 |
| *Anthus cervinus* F (6) | 82.6; 2.1; 79.5–85.5 | 56.1; 1.9; 52.5–58.0 | 14.4; 0.4; 14.0–15.2 | 3.2; 0.1; 3.1–3.4 | 3.2; 0.1; 3.1–3.3 | 20.5; 0.6; 20.0–21.6 | 11.1; 0.9; 9.5–12.1 |
| *Anthus spinoletta blakistoni* M (10) | 93.8; 2.1; 91.5–98.0 | 68.3; 3.2; 64.0–74.5 | 16.7; 0.7; 15.5–17.6 | 3.3; 0.1; 3.1–3.4 | 3.3; 0.1; 3.0–3.4 | 22.5; 1.0; 19.7–23.5 | 9.9; 0.7; 8.6–10.9 |
| *Anthus spinoletta blakistoni* F (6) | 88.8; 3.9; 83.5–93.0 | 65.3; 3.9; 60.0–71.0 | 16.4; 0.5; 15.4–17.0 | 3.3; 0.1; 3.2–3.3 | 3.1; 0.2; 2.9–3.4 | 21.9; 0.8; 20.8–22.8 | 9.7; 0.9; 9.0–11.3 |

**Table S2**. Measurements of the four *madanga ruficollis* specimens and of all species of “small pipits” (*sensu* [1]; except *A. petrosus*, which is very similar to *A. spinoletta*, with which it was previously considered conspecific, see [1]). M – male; F – female; ht – holotype – pt – paratype. Number in parentheses after species name is number of individuals measured. Values are mean, standard deviation and range, in that order.

**Supplementary Figure S1.** Bayesian inference tree of *Madanga ruficollis*, *Amaurocichla bocagii* and a broad selection of passerine birds based on mitochondrial *ND2*, and nuclear *myo*, *ODC* and *CHD1Z* sequence data analysed in four partitions. Values at nodes are posterior probabilities.

**Supplementary Figure S2.** Bayesian inference tree of *Madanga ruficollis*, *Amaurocichla bocagii* and representatives of all clades of *Anthus* and *Motacilla* found in previous studies [2,3], including all “small pipits” (*sensu* [1]; except *A. petrosus*, which was previously considered conspecific with *A. spinoletta*, see [1]. Based on mitochondrial *cytb* and *ND2*, and nuclear *myo*, *ODC* and *CHD1Z* sequence data analysed in seven partitions. Posterior probabilities (PP) and maximum likelihood bootstrap (MLBS) values are shown at the nodes, in this order; * indicates PP 1.00 or MLBS 100%. Unambiguous indels are indicated at the nodes; ^#^, ODC missing for *A. hodgsoni*. ^€^, ODC missing for *A. brachyurus* and *T. tenellus*; ^§^, ODC missing for *A. nyassae*; ^¶^, CHD1Z missing for *Amaurocichla*; ^Ω^, that part of CHD1Z missing from *A. hodgsoni*; insertion in *A. rubescens* 18 bp.

**Supplementary Figure S3.** Single-locus analyses of the same taxa as in Supplementary Figure S2. Posterior probabilities (PP) and maximum likelihood bootstrap (MLBS) values are shown at the nodes; * indicates PP 1.00 or MLBS 100%.

**Supplementary Figure S4.** Chronogram for the same taxa as in Supplementary Figures 2 and 3, based on *cytb* sequences and a relaxed molecular clock (2.1% / million years), inferred by Bayesian inference. Blue bars at nodes represent 95% highest posterior density intervals for the node ages.

**References**

1. Alström P, Mild K. 2003 *Pipits and wagtails of Europe, Asia and North America*: *identification and systematics*. London: Helm/A&C Black; Princeton: Princeton University Press.
2. Voelker G. 1999 Dispersal, vicariance and clocks: historical biogeography and speciation in a cosmopolitan passerine genus (*Anthus*: Motacillidae). *Evolution* **53**, 1536–1552.
3. Alström P, Ödeen A. 2002 Incongruence between mitochondrial DNA, nuclear DNA and non-molecular data in the avian genus *Motacilla*: implications for estimates of species phylogenies. In Alström, P. *Species limits and systematics in some passerine birds*. Ph.D. thesis. Uppsala: Uppsala University.
