# Supplementary material for: Dramatic niche shifts and morphological change in two insular bird species
Source: R Soc Open Sci. 2015 Mar 4;2(3):140364. doi: 10.1098/rsos.140364 (PMC4448822; doi:10.1098/rsos.140364)

Communalities[a]

	Initial	Extraction
wing	1,000	,820
tail	1,000	,798
bill length	1,000	,784
bill depth	1,000	,850
bill width	1,000	,817
tarsus	1,000	,819
hind claw	1,000	,590

Extraction Method: Principal Component Analysis.

a. Only cases for which selection variable = 1 are used in the analysis phase.

Total Variance Explained[a]

Component	Initial Eigenvalues			Extraction Sums of Squared Loadings			Rotation ...	Rotation Sums of Squared ...	
	Total	% of Variance	Cumulative %	Total	% of Variance	Cumulative %	Total	% of Variance	Cumulative %
1	3,998	57,111	57,111	3,998	57,111	57,111	3,281	46,872	46,872
2	1,481	21,150	78,261	1,481	21,150	78,261	2,197	31,388	78,261
3	,796	11,365	89,626						
4	,235	3,351	92,977						
5	,218	3,111	96,089						
6	,178	2,538	98,627						
7	,096	1,373	100,000						

Total Variance Explained[a]

Extraction Method: Principal Component Analysis.

a. Only cases for which selection variable = 1 are used in the analysis phase.

Component Matrix[a,b]

	Component	
	1	2
wing	,842	,334
tail	,781	,434
bill length	,859	,216
bill depth	,742	-,547
bill width	,716	-,551
tarsus	,858	,287
hind claw	-,376	,670

Extraction Method: Principal Component Analysis.

a. 2 components extracted.

b. Only cases for which selection variable = 1 are used in the analysis phase.

Rotated Component Matrix[a,b]

	Component	
	1	2
wing	,890	,166
tail	,892	,050
bill length	,842	,275
bill depth	,336	,859
bill width	,312	,848
tarsus	,879	,215
hind claw	,040	-,767

Extraction Method: Principal Component Analysis.
Rotation Method: Varimax with Kaiser Normalization.

a. Rotation converged in 3 iterations.

b. Only cases for which selection variable = 1 are used in the analysis phase.

Component Transformation Matrix[a]

Component	1	2
1	,846	,534
2	,534	-,846

Extraction Method: Principal Component Analysis.
Rotation Method: Varimax with Kaiser Normalization.

a. Only cases for which selection variable = 1 are used in the analysis phase.

```
GRAPH
 /SCATTERPLOT(BIVAR)=FAC1_1 WITH FAC2_1 BY group
```

```
  /MISSING=LISTWISE.
```

Graph

Notes

**Output Created**		**18-OCT-2013 14:32:20**
**Comments**		
**Input**	**Data**	/Users/Per/Dropbox/Pågående arbeten/Madanga/Measurements/Mått Madanga Anthus.sav
	**Active Dataset**	**DataSet2**
	**Filter**	**<none>**
	**Weight**	**<none>**
	**Split File**	**<none>**
	N of Rows in Working Data File	**176**
**Syntax**		GRAPH /SCATTERPLOT(BIVAR) =FAC1_1 WITH FAC2_1 BY group /MISSING=LISTWISE.
**Resources**	**Processor Time**	**00:00:00,88**
	**Elapsed Time**	**00:00:05,00**

```
[DataSet2] /Users/Per/Dropbox/Pågående arbeten/Madanga/Measurements/Mått Madanga Anthus.sav
```

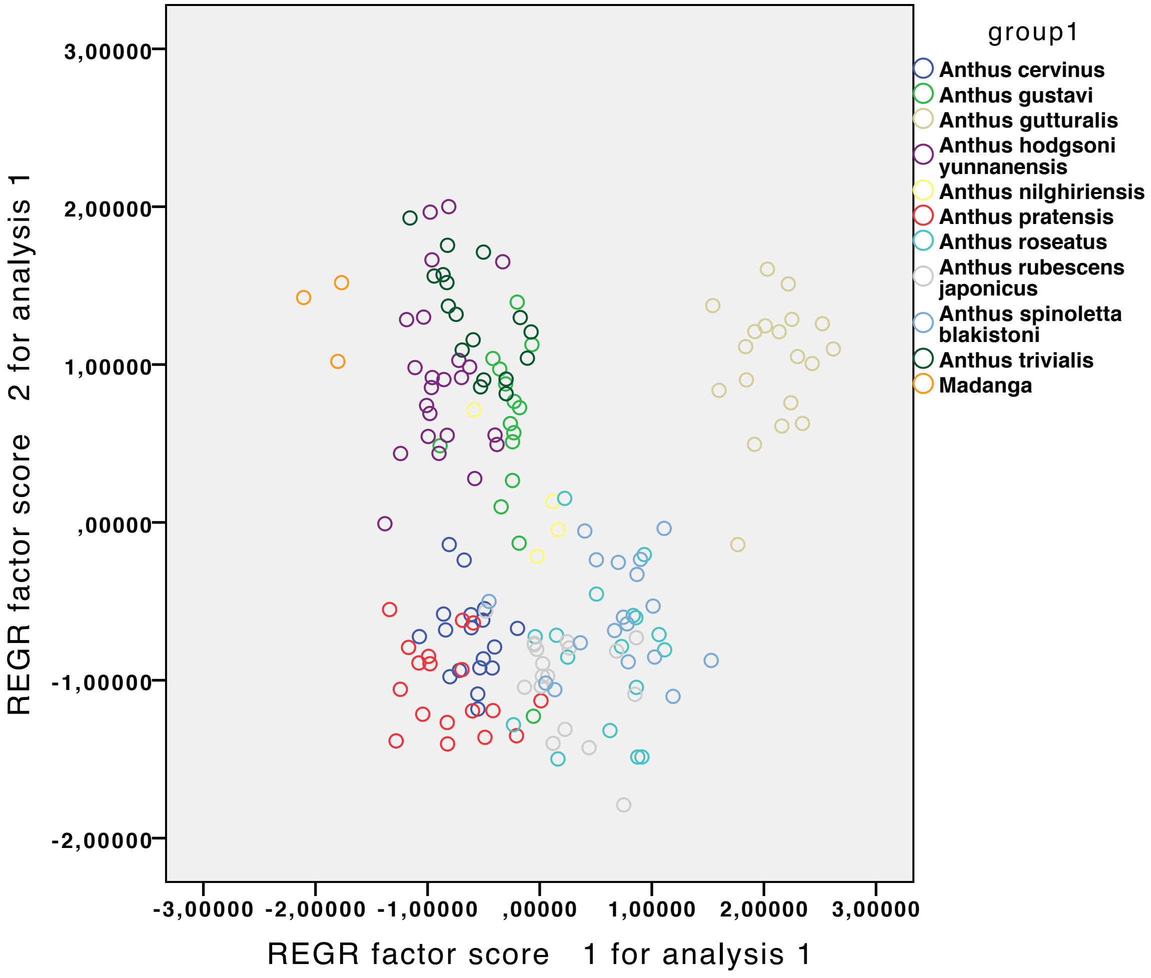

Supplement: Table S3 PCA Madanga.pdf - PCA output [file rsos140364supp7.pdf]
